# Editorial: Using multi-omics to develop new strategies to improve prognosis and immunotherapy outcomes in cancers

**DOI:** 10.3389/fmolb.2023.1190116

**Published:** 2023-06-06

**Authors:** Chenxi Cao, Shuang Chen, Libo Wang, Zaoqu Liu, Xinwei Han

**Affiliations:** ^1^ Department of Interventional Radiology, The First Affiliated Hospital of Zhengzhou University, Zhengzhou, Henan, China; ^2^ Department of Cardiology, The First Affiliated Hospital of Zhengzhou University, Zhengzhou, Henan, China; ^3^ Key Laboratory of Cardiac Injury and Repair of Henan Province, Zhengzhou, Henan, China; ^4^ Center of Reproductive Medicine, The First Affiliated Hospital of Zhengzhou University, Zhengzhou, Henan, China; ^5^ Department of Hepatobiliary and Pancreatic Surgery, The First Affiliated Hospital of Zhengzhou University, Zhengzhou, Henan, China; ^6^ Interventional Institute of Zhengzhou University, Zhengzhou, Henan, China; ^7^ Henan Interventional Therapy and Clinical Research Center, Zhengzhou, Henan, China

**Keywords:** multi-omics, immunotherapy, prognostic signature, molecular subtype, biomarker

The identification and development of novel biomarkers to accurately predict the efficacy of immunotherapy is a critical challenge in the field of tumor immunotherapy. Next-generation sequencing has emerged as a powerful tool for detecting cancer-related molecular alterations on a large scale, refining the molecular classification of cancer, and facilitating research and development of new molecularly targeted drugs and individualized therapeutic strategies (Berger and Mardis, 2018). Further rapid advances in molecular analysis technologies have enabled us to decipher the molecular composition of tumors at a single-cell resolution (Lewis et al., 2021). Subcellular-level spatial genomics, transcriptomics, and proteomics describe cellular interactions between tumor cells and the tumor immune microenvironment (Marx, 2021; Zhao et al., 2022). Multi-omics analysis of biomarkers such as circulating tumor cells provides profound insight into the dynamics of tumor molecular structure during tumor progression and treatment (Corcoran and Chabner, 2018). In addition, functional analysis *in vitro* shed light on the exploration of drug sensitivity in tumor patients (Driehuis, Kretzschmar, and Clevers, 2020). Advancements in multi-omics technologies, such as genomics, transcriptomics, proteomics, metabolomics, and microbiomics, have led to the discovery of numerous biomarkers with high value in clinical practice. These biomarkers enable more accurate diagnoses, more effective treatments, and more precise judgment in disease prognosis for patients (Hasin, Seldin, and Lusis, 2017) (Figure 1). Multi-omics techniques possess tremendous potential in the treatment and prognosis of tumors such as ovarian cancer. They offer promising avenues for the treatment and prognosis of tumors, with potential applications in identifying new biomarkers and improving clinical assessment (Xiao et al., 2022).

**FIGURE 1:**
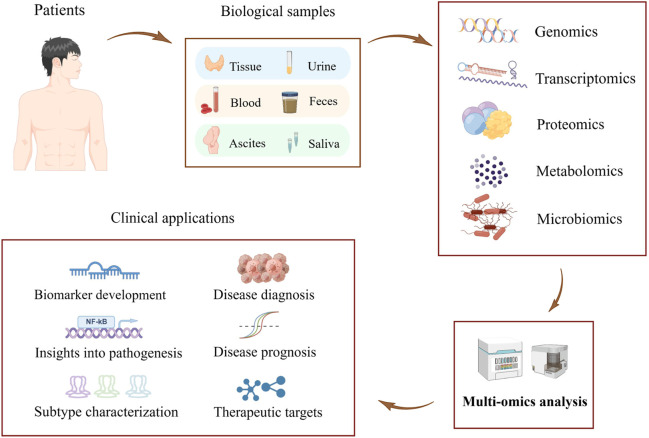
Schematic representation of a multi-omics approach to discovering biomarkers for the early diagnosis of tumors (The figure was created with Figdraw.com).

This Research Topic consisted of four articles authored by 29 experts that aimed to share novel strategies for developing multi-omics, improving immunotherapy efficacy, and enhancing patient prognosis. Two centers in these articles focused on the discovery and validation of biomarkers that could aid in predicting immunotherapy efficacy and patient prognosis, thus providing valuable insights for cancer patients. Among these biomarkers, albumin levels emerged as a strong prognostic indicator for cancer patients treated with immune checkpoint inhibitors (ICI) due to their association with both nutritional and inflammatory status. Guven et al. conducted a comprehensive analysis of 36 studies encompassing 8,406 advanced cancer patients from various databases and found that patients with lower albumin levels had a significantly higher risk of death compared to those with higher levels. Additionally, every 1 g/dL decrease in albumin levels led to a 10% increase in the risk of death, making albumin levels a critical sequential prognostic factor. Another promising biomarker was microbial status before ICI treatment initiation, which could predict patient outcomes. Shoji et al. analyzed the diversity of oral and gut microbiota in 28 non-small cell lung cancer (NSCLC) patients undergoing ICI treatment and revealed that gut microbiota composition significantly differed between responders and non-responders. Conversely, no statistically significant difference in oral microbiota composition was observed, thus further revealing a close relationship between ICI response and gut microbiota diversity in NSCLC patients. Extracellular vesicles (EVs) were broadly classified into three main categories based on their biosynthetic or secretory processes: exosomes, microvesicles/particles/extracellular bodies, and apoptotic vesicles (Yanez-Mo et al., 2015). With the arrival of the “omics era” and a deeper understanding of EVs, Lu et al. discussed a novel class of EV-centered immunotherapies for cancer, summarizing advances in the multi-omic analysis of EVs for the early diagnosis of precancer and hepatocellular carcinoma. Closely related to tumor progression, apoptosis has been recognized as one of the hot spots of research in recent years. IAGsPI and pAGsPI were potential biomarkers to predict the prognosis of patients with renal clear cell carcinoma (Lin et al., 2022). A model based on the pyroptosis-related genes (PRGs) was developed to predict the prognosis of colon adenocarcinoma with excellent performance in external cohorts (Luo et al., 2021). In addition, serving as one of the members of the gastrin family, GSDMD was differentially expressed in most cancers and could perform as a prognostic indicator for adrenocortical carcinoma (ACC) (Qiu, Hu, and Dong, 2021). Gao et al. utilized basic experimental validation and systematic bioinformatics analysis to model the prognosis of PRGs features in ACC patients and analyzed the correlation between immune infiltration and PRGs.

## Conclusion

The Research Topic “*Using Multi-Omics to Develop New Strategies for Improving Prognosis and Immunotherapy Outcomes in Cancer*” serves as a platform for sharing innovative strategies developed through multi-omics to enhance the effectiveness of immunotherapies and improve patient outcomes. These studies integrate data from various layers, providing valuable insights into the molecular structure of tumors and expanding the scope of cancer biology. Although clinical applications of these techniques are still in the early stages, we believe that several of these new approaches will not only advance our understanding of tumor biology but also significantly shape the future of precision cancer therapies.
